# Molecular Epidemiology and Genetic Diversity of *Orientia tsutsugamushi* from Patients with Scrub Typhus in 3 Regions of India

**DOI:** 10.3201/eid2101.140580

**Published:** 2015-01

**Authors:** George M. Varghese, Jeshina Janardhanan, Sanjay K. Mahajan, David Tariang, Paul Trowbridge, John A.J. Prakash, Thambu David, Sowmya Sathendra, O.C. Abraham

**Affiliations:** Christian Medical College, Vellore, India (G.M. Varghese, J. Janardhanan, J.A.J. Prakash, T. David, S. Sathendra, O.C. Abraham);; Indira Gandhi Medical College, Shimla, India (S.K. Mahajan); Dr.H.Gordon Roberts Hospital, Jaiaw, Shillong, India (D. Tariang);; Tufts Medical Center, Boston, Massachusetts, USA (P. Trowbridge)

**Keywords:** scrub typhus, Orientia tsutsugamushi, genetic diversity, molecular epidemiology, 56-kDa TSA, antigenic variation, India, bacteria, acute febrile illness, type-specific antigen, circulation, genotypes, strains

## Abstract

Scrub typhus, an acute febrile illness that is widespread in the Asia-Pacific region, is caused by the bacterium *Orientia tsutsugamushi*, which displays high levels of antigenic variation. We conducted an investigation to identify the circulating genotypes of *O. tsutsugamushi* in 3 scrub typhus–endemic geographic regions of India: South India, Northern India, and Northeast India. Eschar samples collected during September 2010–August 2012 from patients with scrub typhus were subjected to 56-kDa type-specific PCR and sequencing to identify their genotypes. Kato-like strains predominated (61.5%), especially in the South and Northeast, followed by Karp-like strains (27.7%) and Gilliam and Ikeda strains (2.3% each). Neimeng-65 genotype strains were also observed in the Northeast. Clarifying the genotypic diversity of *O. tsutsugamushi* in India enhances knowledge of the regional diversity among circulating strains and provides potential resources for future region-specific diagnostic studies and vaccine development.

## Abstract 2

Clarifying local antigenic diversity is critical for development of region-specific vaccines and diagnostics.

Scrub typhus is a vector-borne, acute febrile illness caused by *Orientia tsutsugamushi*, an obligate intracellular, gram-negative bacterium. Scrub typhus is widespread in the Asia-Pacific region, known as the “tsutsugamushi triangle.” Mite larvae, or chiggers, of the genus *Leptotrombidium* transmit the causative bacteria to humans through their bite. The infection is maintained in nature through transovarial transmission in the vector and a reservoir in small mammals (*1*,*2*). Clinical signs and symptoms of scrub typhus in humans are largely nonspecific, and if infection is not treated promptly and appropriately, it carries a high mortality rate (*3*).

The *Orientia* genome has a high degree of plasticity and is considered to be the most highly repetitive bacterial genome sequenced (*4*). This diversity is a result of high numbers of intragenomic deletions, duplications, and rearrangements with transposable and conjugative elements. These recombinations and rearrangements are unlikely to occur in dead-end hosts, but the details of this process are unclear (*5*). 

Clarifying the epidemiology and genetic diversity of *O. tsutsugamushi* strains is essential to the development of rapid diagnostics and vaccines in disease-endemic areas. These efforts would also help in the early recognition and treatment of the disease. Currently, the most widely used method for strain classification is sequence analysis of the 56-kDa type-specific antigen (TSA), an immunodominant outer membrane protein unique to *O. tsutsugamushi*. With an open reading frame (ORF) of ≈1,600 bp, the 56-kDa TSA contains 516–541 amino acids and is involved in host cell invasion through the binding of fibronectin (*6*). Four hypervariable domains in this region, variable domains (VD) I–IV, are responsible for the large degree of antigenic variation in this gene. The direct interaction with the host, uniqueness to *O. tsutsugamushi*, and high level of variability make this protein an attractive target for studying the genetic variation among strains. This region is also highly immunogenic, making it a potential candidate as a vaccine target. 

The process of conventional serotyping was a complex procedure, requiring reference serum samples and antigens, and is of limited use today. Greater diversity among the strains has been revealed by using molecular genotyping methods. Antigenic variations in *O. tsutsugamushi* from patients and rodents in different scrub typhus–endemic regions have been reported by testing using the 56-kDa TSA, which has led to identification of several new subtypes (*1*), such as Japanese Gilliam, Japanese Karp, Kawasaki, Kuroki, and Shimokoshi, in addition to the previously described prototypes Karp, Kato, and Gilliam (*7*,*8*). 

Given the broad endemicity of scrub typhus in the Asia-Pacific region and variations in clinical manifestations that may be attributable to strain variation, thorough investigation into the regional distribution of genotypes is warranted. This study was conducted to identify the circulating 56-kDa antigen genotypes in 3 scrub typhus–endemic geographic regions of India: South India, Northern India, and Northeast India.

## Methods

### Study Population

All patients who sought care for fever and suspected scrub typhus at the 3 recruiting centers (Christian Medical College in Vellore, Indira Gandhi Medical College in Shimla, and the Dr. H. Gordon Roberts Hospital in Shillong) during September 2010–August 2012 were evaluated. A detailed clinical assessment of each patient for the signs and symptoms of scrub typhus, including a careful search for an eschar and basic laboratory studies, was documented by using a standardized form. Additional investigations, including blood cultures, quantitative buffy coat (testing for malarial parasites), abdominal ultrasound, and serologic testing for the causative agents of leptospirosis and dengue, were performed to rule out other common infections. Detection of *O. tsutsugamushi* was performed by using the Scrub Typhus Detect IgM ELISA (InBios International, Inc., Seattle, WA, USA) according to the manufacturer’s instructions; an optical density (OD) >0.5 was considered positive. De-roofed eschar samples were collected and stored in absolute alcohol at −70°C until DNA extraction. Patients with clinical illness compatible with scrub typhus, including an eschar and positive results for serum samples testing by IgM ELISA, were included in the study. The study was approved by the Institutional Review Board and Ethics Committee of Christian Medical College, and informed consent was obtained from all patients.

### DNA Amplification and Sequence Analysis

DNA was extracted from the homogenized eschar samples by using the QIAamp DNA Mini Kit (QIAGEN GmbH, Hilden, Germany) according to the manufacturer’s instructions. A standard PCR, targeting the 56-kDa protein, was performed as described by using the primers OtsuF: 5′-AATTGCTAGTGCAATGTCTG-3′ and OtsuR: 5′-GGCATTATAGTAGGCTGAG-3′ (Sigma Aldrich, Bangalore, India) (*9*). This region encompasses ≈410 bp and contains the VD I–III hypervariable regions. The PCR products were purified by using the QIAquick PCR Purification Kit (QIAGEN) according to the manufacturer’s instructions; products were then subjected to a sequencing reaction using the BigDye Terminator Mix (Applied Biosystems, Foster City, CA, USA) and subsequent automatic sequencing using the ABI 310 Genetic Analyzer (Applied Biosystems). PCR and sequencing reactions were carried out in the Infectious Diseases Research laboratories at Christian Medical College in Vellore. 

The sequences obtained were identified by comparison with sequences available in GenBank by using BLAST (http://blast.ncbi.nlm.nih.gov). All sequences obtained were submitted to GenBank (accession nos. KC153061–KC153085 and KF777306–KF777328 for samples from Vellore; KF777265–KF777290, KF777292, KF777294–KF777305 for samples from Shimla; and KF777329–KF777357 for samples from Shillong). Phylogenetic and molecular evolutionary analyses were conducted by using MEGA5 (*10*). The study sequences and reference sequences downloaded from GenBank were aligned by using ClustalW (http://www.clustal.org) and trimmed to the appropriate size. A phylogenetic tree with 1,000 bootstrap replications was constructed by using neighbor-joining methods with distances calculated by the maximum composite likelihood. Statistical analysis was performed by using SPSS software version 16.0 for Windows (SPSS IBM, Armonk, NY, USA). Descriptive data are given as mean (SD) or median (range).

## Results

A total of 263 eschar samples were obtained from patients with confirmed scrub typhus from the 3 study sites: 95 from Vellore in South India, 72 from Shimla in Northern India, and 96 from Shillong in Northeast India. Of the patients from whom these samples were collected, 115 (43.7%) were male and 148 (56.3%) female. Mean patient age was 40 ± 12 years.

Of the 263 samples, 209 (79.5%) were successfully amplified by using 56-kDa conventional PCR. Of the 209 amplified samples, 130 (62.2%) yielded good sequencing reads (58 from Vellore, 42 from Shimla, and 30 from Shillong). These reads were then used for further data analysis.

### Sequence Analysis

Sequence analysis revealed that Kato-like strains predominated; 80 (61.5%) of the 130 total samples analyzed yielded Kato-like strains, followed by Karp-like strains with 36 (27.7%) samples and Gilliam and Ikeda strains with 3 (2.3%) samples each. Four (3.1%) samples from Shillong were similar to Neimeng-65, and 4 (3.1%) from Shimla were similar to IHS-II. The Karp-like amplicons showed a nucleotide similarity of 88% to 95% to the Karp reference strain (GenBank accession no. AY956315), and the nucleotide similarity for Kato-like strains to the US Centers for Disease Control and Prevention (CDC) Kato reference strain (GenBank accession no. AY836148) ranged from 90% to 94%. Most sequences showed 90%–96% similarity to Hualien strains from Taiwan. 

**Figure 1:**
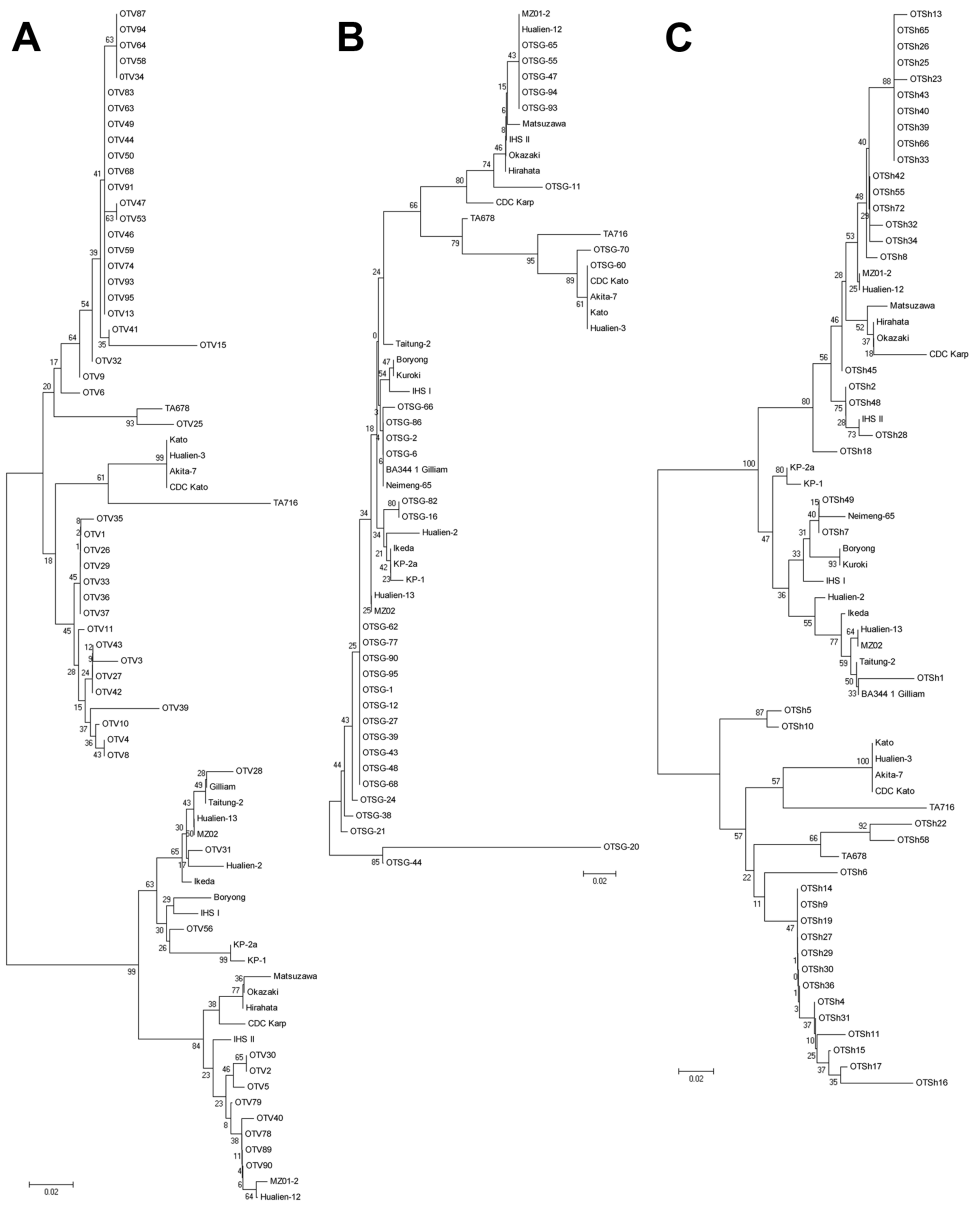
Phylogenetic distribution of *Orientia tsutsugamushi*isolates from scrub typus patients in Vellore (A), Shillong (B), and Shimla (C), India, September 2010–August 2012. Isolates were identified on the basis of the 56-kDa TSA gene. Evolutionary history was inferred by using the neighbor-joining method. The percentage of replicate trees in which the associated taxa clustered together in the bootstrap test (1,000 replicates) is shown next to the branches. The tree is drawn to scale, with branch lengths in the same units as those of the evolutionary distances used to infer the phylogenetic tree. Evolutionary distances were computed by using the maximum composite likelihood method (*10*). Scale bars indicate base substitutions per site. Sequences identified in this study were deposited in GenBank under accession nos. KC153061–KC153085 and KF777306–KF777328 (OTV), KF777329–KF777357 (OTSG), and KF777265–KF777290, KF777292, and KF777294– KF777305 (OTSh). OTV, *O. tsutsugamushi* from Vellore; OTSG, *O. tsutsugamushi* from Shillong; OTSh, *O. tsutsugamushi* from Shimla.

The distributions of genotypes by center are displayed in the Table. Among the 58 samples analyzed from Vellore, 45 (77.5%) were Kato-like, and most of those (35, 77.7%) showed 92%–96% sequence similarity to the Hualien-3 strain from Taiwan. Two Kato-like amplicons were 94% similar to another strain from Taiwan (GenBank accession no. KM0607b), and 4 others were 92%–95% similar to strain TA678 from South Korea. The remaining showed similarities ranging from 92% to 95% to various strains such as MZ02, KM06, and HL03 from Taiwan. Eleven (18.9%) samples from Vellore yielded Karp-like amplicons, and these showed 95%–98% sequence similarities to the clone T0122244_KH from Cambodia and Vietnam. One Gilliam strain found in Vellore showed 99% similarity to the BA344_1 Gilliam strain from Thailand (GenBank accession no. JN587265). One Ikeda strain was also found in Vellore. Phylogenetic analysis of the Vellore strains with reference strains showed 2 distinct clusters (Figure 1, panel A); one of the clusters included 11 strains from Vellore along with the Karp reference strains Taiwan CDC Karp, Kp-1, and Kp-2a.

**Table:** Geographic distribution of *Orientia tsutsugamushi* genotypes in 3 regions of India, September 2010–August 2012

Genotype	Vellore, n = 58	Shimla, n = 42	Shillong, n = 30	Total, n = 130
Kato-like	45 (77.5)	18 (42.8)	17 (56.6)	80
Karp-like	11 (18.9)	18 (42.8)	7 (23.3)	36
Gilliam	1 (1.7)	0	2 (6.6)	3
Ikeda	1 (1.7)	2 (4.7)	0	3
IHS-II	0	4 (9.5)	0	4
Neimeng-65	0	0	4 (13.3)	4

Kato-like sequences also predominated among the 30 analyzed sequences from Shillong; 17 (56.6%) samples belonged to this strain. These genotypes, however, were generally most similar (94%–95%) to the Hualien-13 strain from Taiwan. Some were also highly similar (98%) to the clone T1116018_KH from Cambodia and Vietnam, and 1 was 99% similar to the reference strain Taiwan CDC Kato. Seven samples (23.3%) from Shillong were Karp-like strains, 2 (6.6%) were Gilliam strains, and 4 (13.3%) were Neimang-65 strains. Two major clusters were observed on phylogenetic analysis, with a few of the strains clustering with the Hualien-13 strains and the remaining strains scattered in a second, larger cluster with several subgroups (Figure 1, panel B).

Of the 42 sequences analyzed from Shimla, 18 (42.8%) were Kato-like, and most showed 94%–96% similarity to the Hualien-3 strain. The samples from Shimla also included 18 (42.8%) Karp-like samples, which were most similar to the MZ01 strain obtained from chiggers from Taiwan. Two (4.7%) samples were found to be of the Ikeda strain and 4 (9.5%) of the IHS-II strain. Two distinct clusters with subgroups were observed on phylogenetic analysis (Figure 1, panel C).

**Figure 2:**
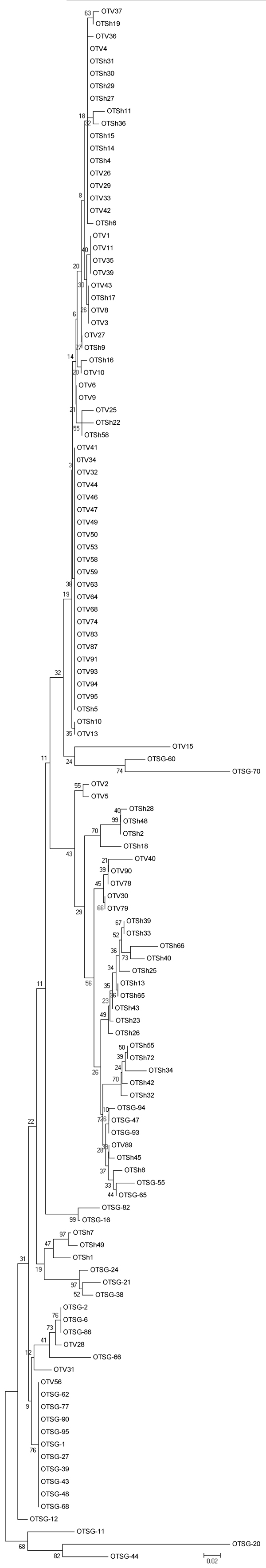
Phylogenetic distribution of representative *Orientia tsutsugamushi*isolates from scrub typus patients in India, September 2010–August 2012. Isolates were identified on the basis of the 56-kDa TSA gene. The evolutionary history was inferred by using the neighbor-joining method. The percentage of replicate trees in which the associated taxa clustered together in the bootstrap test (1,000 replicates) is shown next to the branches. The tree is drawn to scale, with branch lengths in the same units as those of the evolutionary distances used to infer the phylogenetic tree. The evolutionary distances were computed by using the maximum composite likelihood method (*10*). Scale bar indicates base substitutions per site.

A comparison of sequences obtained in this study revealed that the samples from the 3 centers clustered separately on the neighbor-joining tree. The Vellore strains formed a distant clade, whereas the Shillong and Shimla strains clustered more closely together (Figure 2).

## Discussion

Scrub typhus has been recognized as a major cause of undifferentiated acute febrile illness in India (*11*). Previous studies have reported the prevalence of scrub typhus in Vellore and Shimla (*12**–**15*), whereas reports from Shillong are few and fairly recent (*16*,*17*). Scrub typhus has been reported from states throughout India: Kerala, Karnataka, Andhra Pradesh, and Tamil Nadu in South India; Bihar, Maharashtra, Jammu Kashmir, Himachal Pradesh, Uttaranchal, and Rajasthan in Northern India; and Meghalaya, Sikkim, and West Bengal in Northeast India (*9*,*12*,*18**–**24*). Despite the broad effect of scrub typhus in India, little genotyping has been performed, with results available only from Himachal Pradesh (*9*). Similarly, although scrub typhus has been reported from neighboring countries, such as Pakistan, Myanmar, and Nepal, genotype data are lacking from these regions, making comparisons difficult (*25*,*26*). 

In this study, we identified the circulating genotypes of *O. tsutsugamushi* in 3 scrub typhus–endemic regions in India by analyzing a variable portion of the 56-kDa antigen gene. Overall, 80 (61.5%) of 130 samples were Kato-like strains, which have previously been found by serologic testing and genotyping in other locations, primarily Japan and Cambodia, but not as a predominant strain (*1*,*4*,*27*,*28*). The strains we identified were largely similar to the Hualien-3 reference sample, which was originally reported from Taiwan (*6*). However, some Kato-like strains showed closer similarity to the TA678 strain from South Korea. In Shillong, the Kato-like strains were most similar to another Taiwan strain, Hualien-13. Other Kato-like strains that bore a closer resemblance to the clone T1116018_KH from Cambodia and Vietnam were also found in Shillong. 

The highest proportion of Kato-like strains (77.5%) was found at Vellore in South India, with progressively smaller proportions of this strain seen as locations progressed to the north. The highest proportion of Kato-like strains in a single region (21.5%) was previously reported from Cambodia (*4*); South India’s climate is more similar to Cambodia’s climate than to Northern India’s climate, so temperature or other environmental factors may play a role in the selecting for this strain. However, most of the Kato-like strains we found, particularly from Vellore, resembled strains from Taiwan rather than Cambodia, which could argue against a climate-based selection process. Kato-like strains in Shillong, which is the geographically closest of our sites to Cambodia, resemble the strains found in Southeast Asia.

Karp-like strains, although less common than Kato-like in our study, are more widespread globally, reported by serologic testing and genotyping in Southeast Asia (Thailand, Vietnam, Cambodia, the Philippines, and Malaysia), Japan, southern China, Taiwan, Mongolia, Oceania, and Australia; these strains also are cited as the most common circulating strain in several Southeast Asia countries and in Japan (*1*,*4*,*27**–**30*). By serologic testing, these strains have been previously reported in India and in Pakistan just to the north (*1*). In our study, Karp-like strains were at their highest proportion at Shimla in the northern part of the country (equal to the amount of Kato-like strains), with progressively smaller proportions of this grouping successively farther south. Most Karp-like strains from Vellore showed high sequence similarity to strains previously found in Cambodia and Vietnam; strains in 5 samples showed a high degree of similarity to the Ld-1a strain (GenBank accession no. JN415541), which was reported from trombiculid mites in Thailand. Unfortunately, sequencing data were not sufficient to assess for similarities in the Karp-like samples from Shillong or Shimla.

Our study also identified Gilliam-like strains in Vellore and Shillong; all of these sample strains were similar to the BA244_1 strain previously found in mites from Thailand. Gilliam strains have been reported in a variety of locations, including Japan, Korea, China, Taiwan, Thailand, Russia, Tajikistan, and Northern India (*1*,*29*,*31**–**35*). All of our samples, including those from Shillong in Northeast India, were most similar to genotypes previously seen in Thailand, not to the Gilliam-like strain from Myanmar (GenBank accession no. DQ286233) that was previously reported in Northern India. In addition, we isolated Neimeng-65 strains in Shillong. This finding is not surprising in Northeastern India, considering the proximity of the area to the border of China, where Neimeng-65 was originally reported in rodents from Inner Mongolia and Xinjian (GenBank accession no. DQ514319). We also found Ikeda-like strains from Vellore and Shimla; these strains were previously reported primarily from Japan (*27*).

Initial molecular descriptions of *O. tsutsugamushi* in India were reported in Shimla, where 2 new genotypes, IHS-I (GenBank accession no. DQ286233DQ530440) and IHS-II (GenBank accession no. DQ286233DQ530441), were identified based on partial 56k-Da TSA sequences (*9*). Strains in 4 samples, all from Shimla, in this study showed the highest nucleotide similarity to the IHS-II strain. Seerangayee strains and the Kuroki strain of the Boryong cluster, previously reported in India (*1*,*34*), were not identified in our study. Other groupings, such as Kawasaki, TA673, TA716, and Japanese Gilliam, were also not observed.

Our study has limitations. Not all scrub typhus patients have an eschar, so we could not run PCR testing on samples from all patients. If some strains are more likely than others to produce an eschar, some strains may have been over- or underrepresented in our study. In addition, not all eschar samples were amenable to good sequencing, which could also have skewed our results.

In summary, we identified the circulating genotypes of *O. tsutsugamushi* in 3 scrub typhus–endemic regions of India. Kato-like strains were found to be predominant in the South and Northeast, whereas an equal prevalence of Karp-like and Kato-like strains was observed in Northern India. The prevalence of antigenic diversity can have wider implications in vaccine development; also, a potential association between strain variation and pathogenicity of scrub typhus has reported in mice studies (*36*,*37*). In addition, targeted serodiagnosis will require further knowledge of this variability. Thus, an accurate picture of the local antigenic diversity will be essential for the development of region-specific vaccines and diagnostics.

## References

[1] Kelly DJ, Fuerst PA, Ching WM, Richards AL. Scrub typhus: the geographic distribution of phenotypic and genotypic variants of *Orientia tsutsugamushi.* Clin Infect Dis. 2009;48(Suppl 3):S203–30 . 10.1086/596576 19220144

[2] Wongprompitak P, Anukool W, Wongsawat E, Silpasakorn S, Duong V, Buchy P, Broad-coverage molecular epidemiology of *Orientia tsutsugamushi* in Thailand. Infect Genet Evol. 2013;15:53–8. 10.1016/j.meegid.2011.06.008 21712103

[3] Mathai E, Lloyd G, Cherian T, Abraham OC, Cherian AM. Serological evidence for the continued presence of human rickettsioses in southern India. Ann Trop Med Parasitol. 2001;95:395–8 . 10.1080/00034980120065804 11454249

[4] Cho NH, Kim HR, Lee JH, Kim SY, Kim J, Cha S, The *Orientia tsutsugamushi* genome reveals massive proliferation of conjugative type IV secretion system and host-cell interaction genes. Proc Natl Acad Sci U S A. 2007;104:7981–6. 10.1073/pnas.0611553104 17483455 PMC1876558

[5] Duong V, Mai TT, Blasdell K. Lo le V, Morvan C, Lay S, et al. Molecular epidemiology of *Orientia tsutsugamushi* in Cambodia and Central Vietnam reveals a broad region-wide genetic diversity. Infect Genet Evol. 2013;15:35–42. 10.1016/j.meegid.2011.01.004 21241829

[6] Lin PR, Tsai HP, Tsui PY, Weng MH, Kuo MD, Lin HC, Genetic typing, based on the 56-kilodalton type specific antigen gene, of *Orientia tsutsugamushi* strains isolated from chiggers collected from wild-caught rodents in Taiwan. Appl Environ Microbiol. 2011;77:3398–405 . 10.1128/AEM.02796-10 21441323 PMC3126458

[7] Nakayama K, Kurokawa K, Fukuhara M, Urakami H, Yamamoto S, Yamazaki K, Genome comparison and phylogenetic analysis of *Orientia tsutsugamushi* strains. DNA Res. 2010;17:281–91. 10.1093/dnares/dsq018 20682628 PMC2955711

[8] Yang H-H, Huang I-T, Lin C-H, Chen T-Y, Chen L-K. New genotypes of *Orientia tsutsugamushi* isolated from humans in eastern Taiwan. PLoS ONE. 2012;7:e46997. 10.1371/journal.pone.0046997 23071693 PMC3468442

[9] Mahajan SK, Rolain JM, Kashyap R, Bakshi D, Sharma V, Prasher BS, Scrub typhus in Himalayas. Emerg Infect Dis. 2006;12:1590–2. 10.3201/eid1210.051697 17176580 PMC3290934

[10] Tamura K, Peterson D, Peterson N, Stecher G, Nei M, Kumar S. MEGA5: molecular evolutionary genetics analysis using maximum likelihood, evolutionary distance, and maximum parsimony methods. Mol Biol Evol. 2011;28:2731–9. 10.1093/molbev/msr121 21546353 PMC3203626

[11] Isaac R, Varghese GM, Mathai E, Manjula J, Joseph I. Scrub typhus: prevalence and diagnostic issues in rural southern India. Clin Infect Dis. 2004;39:1395–6. 10.1086/424748 15494919

[12] Chrispal A, Boorugu H, Gopinath KG, Prakash JA, Chandy S, Abraham OC, Scrub typhus: an unrecognized threat in South India—clinical profile and predictors of mortality. Trop Doct. 2010;40:129–33. 10.1258/td.2010.090452 20360426

[13] Mathai E, Rolain JM, Verghese GM, Abraham OC, Mathai D, Mathai M, Outbreak of scrub typhus in southern India during the cooler months. Ann N Y Acad Sci. 2003;990:359–64 . 10.1111/j.1749-6632.2003.tb07391.x 12860654

[14] Sharma A, Mahajan S, Gupta ML, Kanga A, Sharma V. Investigation of an outbreak of scrub typhus in Himalayan region of India. Jpn J Infect Dis. 2005;58:208–10 . 16116251

[15] Kumar K, Saxena VK, Thomas TG, Lal S. Outbreak investigation of scrub typhus in Himachal Pradesh (India). J Commun Dis. 2004;36:277–83 . 16506551

[16] Dass R, Deka NM, Duwarah SG, Barman H, Hoque R, Mili D, Characteristics of pediatric scrub typhus during an outbreak in the North Eastern region of India: peculiarities in clinical presentation, laboratory findings and complications. Indian J Pediatr. 2011;78:1365–70 . 10.1007/s12098-011-0470-5 21630069

[17] Goswami D, Hing A, Das A, Lyngdoh M. Scrub typhus complicated by acute respiratory distress syndrome and acute liver failure: a case report from Northeast India. Int J Infect Dis. 2013;17:e644–5. 10.1016/j.ijid.2012.12.023 23402799

[18] Saifudheen K, Kumar KG, Jose J, Veena V, Gafoor VA. First case of scrub typhus with meningoencephalitis from Kerala: an emerging infectious threat. Ann Indian Acad Neurol. 2012;15:141–4. 10.4103/0972-2327.95002 PMC3345595 22566732

[19] Boorugu H, Dinaker M, Roy ND, Jude JA. Reporting a case of scrub typhus from Andhra Pradesh. J Assoc Physicians India. 2010;58:520 . 21189708

[20] Batra HV. Spotted fevers and typhus fevers in Tamil Nadu. Indian J Med Res. 2007;126:101–3 . 17932432

[21] Viswanathan S, Muthu V, Iqbal N, Remalayam B, George T. Scrub typhus meningitis in South India—a retrospective study. PLoS ONE. 2013;8:e66595 . 10.1371/journal.pone.0066595 23799119 PMC3682970

[22] Althaf A, Kumar KK, Suni KA, Farook MU. A study on scrub typhus in a tertiary care hospital. Kuwait Med J. 2008;3:11–4.

[23] Khan SA, Dutta P, Khan AM, Topno R, Borah J, Chowdhury P, Re-emergence of scrub typhus in northeast India. Int J Infect Dis. 2012;16:e889–90. 10.1016/j.ijid.2012.05.1030 22796321

[24] Gurung S, Pradhan J, Bhutia PY. Out break of scrub typhus in North East Himalayan region- Sikkim: an emerging threat. Indian J Med Microbiol. 2013;31:72–4. 10.4103/0255-0857.108729 23508434

[25] Murdoch DR, Woods CW, Zimmerman MD, Dull PM, Belbase RH, Keenan AJ, The etiology of febrile illness in adults presenting to Patan hospital in Kathmandu, Nepal. Am J Trop Med Hyg. 2004;70:670–5 . 15211012

[26] World Health Organization. Frequently asked questions: scrub typhus [cited 2013 Oct 1]. http://www.searo.who.int/entity/emerging_diseases/CDS_faq_Scrub_Typhus.pdf

[27] Enatsu T, Urakami H, Tamura A. Phylogenetic analysis of *Orientia tsutsugamushi* strains based on the sequence homologies of 56-kDa type-specific antigen genes. FEMS Microbiol Lett. 1999;180:163–9. 10.1111/j.1574-6968.1999.tb08791.x 10556707

[28] Ohashi N, Nashimoto H, Ikeda H, Tamura A. Diversity of immunodominant 56-kDa type-specific antigen (TSA) of *Rickettsia tsutsugamushi:* sequence and comparative analyses of the genes encoding TSA homologues from four antigenic variants. J Biol Chem. 1992;267:12728–35 . 1618776

[29] Manosroi J, Chutipongvivate S, Auwanit W, Manosroi A. Determination and geographic distribution of *Orientia tsutsugamushi* serotypes in Thailand by nested polymerase chain reaction. Diagn Microbiol Infect Dis. 2006;55:185–90. 10.1016/j.diagmicrobio.2006.01.014 16626907

[30] Lu HY, Tsai KH, Yu SK, Cheng CH, Yang JS, Su CL, Phylogenetic analysis of 56-kDa type-specific antigen gene of *Orientia tsutsugamushi* isolates in Taiwan. Am J Trop Med Hyg. 2010;83:658–63 . 10.4269/ajtmh.2010.09-0608 20810835 PMC2929066

[31] Tamura A, Yamamoto N, Koyama S, Makisaka Y, Takahashi M, Urabe K, Epidemiological survey of *Orientia tsutsugamushi* distribution in field rodents in Saitama Prefecture, Japan, and discovery of a new type. Microbiol Immunol. 2001;45:439–46. 10.1111/j.1348-0421.2001.tb02643.x 11497219

[32] Qiang Y, Tamura A, Urakami H, Makisaka Y, Koyama S, Fukuhara M, Phylogenetic characterization of *Orientia tsutsugamushi* isolated in Taiwan according to the sequence homologies of 56-kDa type-specific antigen genes. Microbiol Immunol. 2003;47:577–83. 10.1111/j.1348-0421.2003.tb03420.x 14524618

[33] Seong SY, Park SG, Huh MS, Jang WJ, Choi MS, Chang WH, T-track PCR fingerprinting for the rapid detection of genetic polymorphism. FEMS Microbiol Lett. 1997;152:37–44. 10.1111/j.1574-6968.1997.tb10406.x 9228768

[34] Bakshi D, Singhal P, Mahajan SK, Subramaniam P, Tuteja U, Batra HV. Development of a real-time PCR assay for the diagnosis of scrub typhus cases in India and evidence of the prevalence of new genotype of *O. tsutsugamushi.* Acta Trop. 2007;104:63–71. 10.1016/j.actatropica.2007.07.013 17870041

[35] Blacksell SD, Luksameetanasan R, Kalambaheti T, Aukkanit N, Paris DH, McGready R, Genetic typing of the 56-kDa type-specific antigen gene of contemporary *Orientia tsutsugamushi* isolates causing human scrub typhus at two sites in north-eastern and western Thailand. FEMS Immunol Med Microbiol. 2008;52:335–42. 10.1111/j.1574-695X.2007.00375.x 18312580

[36] Kim DM, Yun NR, Neupane GP, Shin SH, Ryu SY, Yoon HJ, Differences in clinical features according to Boryoung and Karp genotypes of *Orientia tsutsugamushi.* PLoS ONE. 2011;6:e22731. 10.1371/journal.pone.0022731 21857951 PMC3156117

[37] Chu H, Park SH, Kim EJ, Hwang KJ, Shim SK, Park S, Phylogenetic clustering of 4 prevalent virulence genes in *Orientia tsutsugamushi* isolates from human patients. J Microbiol. 2010;48:124–8. 10.1007/s12275-009-0267-7 20221740

